# Thermotolerance thresholds of the photosystem II increase with elevation in conifer species in the Spanish Pyrenees

**DOI:** 10.1111/plb.70260

**Published:** 2026-07-13

**Authors:** N. Kunert, E. Düsterhöft

**Affiliations:** ^1^ Tropical and Functional Plant Ecology University of Bayreuth Bayreuth Germany

**Keywords:** Heat‐stress, juniper, pine, precipitation, temperature gradient

## Abstract

- *In vitro*‐measured photosystem II (PSII) thermotolerance, indicated by the temperature‐dependent decline of the quantum use efficiency of PSII, has been broadly applied to evaluate plants' ability to deal with future climate warming. Nonetheless, deriving PSII thermotolerance thresholds, such as T5, T15 and T50, representing 5%, 15% and 50% temperature‐dependent decline in quantum use efficiency, has rarely been tested against distributional ranges and associated environmental variables.
- Therefore, we evaluate the predictive power of these three PSII thermotolerance thresholds, obtained from seven temperate conifer species in the Spanish Pyrenees, in association with their respective distributional pattern in regard to altitude and accompanying environmental variables.
- Both T5 and T15 correlated with the mean altitudinal distribution, with species growing in higher elevations exhibiting higher PSII thermotolerance thresholds compared to species in the lowlands. The thresholds were negatively correlated with the mean annual temperature while displaying a positive correlation with the mean annual precipitation. No correlation was found between T50 and elevational as well as environmental gradients. Both T5 and T15 indicate an initial decline of the quantum use efficiency.
- We speculate that plants of greater altitudes, by being exposed to larger temperature fluctuations, have a greater need for higher PSII thermotolerance. As climate change‐induced heat stress intensifies, high‐altitude conifer species might be more resistant to increasing temperatures than previously thought.

## Graphical abstract

*In vitro*‐tested photosystem II thermotolerance of seven temperate conifers indicated higher thermal thresholds in species growing in high elevations compared to those in the lowlands. Higher thermotolerance might be explained by the adaptation of species in higher elevations to extreme diurnal temperature fluctuations.

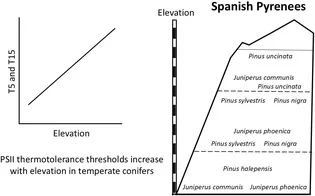

## INTRODUCTION

Temperature extremes in form of heatwaves have become a new reality with ongoing climate change and are predicted to become even more intense and frequent in the near future. Heatwaves result in increased tree respiratory activities, often accompanied by a reduction in photosynthesis (Duarte *et al*. 2016) and significant damage of the photosystem II (PSII; Kunert *et al*. 2026), thereby negatively affecting physiological performance of plants (Feeley *et al*. 2020). In particular, the *in vitro* assessment of photosystem II (PSII) sensitivity to thermal stress has become a common method to estimate leaf‐level heat responses (Neuner & Buchner 2023; Zhang *et al*. 2024). Approaches assessing thermal PSII sensitivity include mainly the measurement of two key parameters of chlorophyll fluorescence. The first one is to assess the temperature‐dependent change in minimal fluorescence of dark‐adapted leaves (*F*_0_). *F*_0_ abruptly rises when the temperature threshold is surpassed. The interception two linear regression, the first forced through of the segment of constant *F*_0_ until its rise and second through the segment of rising *F*_0_, describes the critical temperature (T_crit_; Frolec *et al*. 2008; Zhu *et al*. 2024; Winter *et al*. 2025). Confusingly, T_crit_ is also used to describe important points on the temperature‐dependent function of the second key parameter, namely the maximum quantum yield of PSII photochemistry in dark‐adapted state (*F*_*v*_/*F*_*m*_). Commonly, three metrics are extracted from the function describing the temperature‐dependent decline in *F*_*v*_/*F*_*m*_. The first one is the breaking point temperature (T5), which is the temperature when the maximum quantum use efficiency declines by 5%, further the temperature (T15) when the quantum use efficiency declines by 15%, and lastly the temperature at which 50% of the *F*_*v*_/*F*_*m*_ is lost (T50). Both T5 and T15 are often used analogously to T_crit_ in the literature (T5: Slot *et al*. 2021; Winter 2024; T15: Zhang *et al*. 2024; Manzi *et al*. 2025). All four metrics, T_crit_, T5, T15 and T50, are supposed to predict a functional impairment resulting in significant necrotic leaf tissue (Krause *et al*. 2010; O'sullivan *et al*. 2017; Kunert *et al*. 2026).

The metrics estimated using the above‐described methods are assumed to be largely species‐specific; however, species growing in warmer biomes are generally found to have a higher thermotolerance compared to species of colder biomes (Zhu *et al*. 2018). This apparent trend of thermotolerance displayed by species along latitudinal temperature gradients seems to persist on regional elevational gradients as well (Feeley *et al*. 2020; Slot *et al*. 2021). For example, in the study by Slot *et al*. (2021), leaf‐level thermotolerance of tropical trees, assessed *via* chlorophyll *a*‐fluorescence, was observed to be higher at about sea level than at 750 m above sea level. In the same study, T50 values were significantly higher at warmer low elevation sites. Similarly, the T50 values in tropical montane tree species declined with increasing altitude along a 2,500‐m elevational gradient in the Andes (Feeley *et al*. 2020). In contrast, surprising results were provided by Hernández *et al*. (2025) who found a reverse trend. Herbaceous plants growing in higher altitudes showed higher thermotolerance in comparison with plants of lower elevations. The logical reasoning behind this contradicting finding is that high‐altitude herbaceous species are exposed to a variety of stressors such as high irradiation and high temperatures during the day but are exposed to freezing temperatures during the night. Hernández *et al*. (2025) assumed that those plants developed some sort of cross‐tolerance against various stressors, including higher thermotolerance. Moreover, Neuner & Buchner (2023) reported T50 values for several coniferous tree species, a dwarf shrub and an herbaceous plant, inhabiting the Austrian Alps. In the subalpine to the nival zone, T50 varied between 44.4 °C and 48.4 °C. We found that the observed thermotolerance thresholds for those coniferous tree species in higher elevations (Neuner & Buchner 2023) did not diverge much to reported T50 values of the same species going in the lowlands (Münchinger *et al*. 2023). Münchinger *et al*. (2023) investigated the same species in the vicinity of the northern alps at approximately 590 m above sea level ranged between 45.2 °C and 47.2 °C. Hence, by comparing the findings of these two studies, it does not appear that there is not much of a difference in thermal threshold values between coniferous trees growing in lower, compared to higher altitudes. Nevertheless, studies directly evaluating the PSII thermotolerance of temperate coniferous tree species along elevation gradients are currently absent from the literature.

In this study, we wanted to test (i) whether there is a relationship between the PSII thermotolerance of different conifer species along an elevational gradient, and (ii) whether a possible variation in the PSII thermotolerance can be explained by differences in climatic variables. Therefore, we established temperature–response curves of the photosynthetic quantum use efficiency (*F*_*v*_/*F*_*m*_) for seven conifer species (three juniper species and four pine species) in the Spanish Pyrenees. We extracted the above‐presented three metrics, namely T5, T15 and T50, from the temperature–response curves. The seven conifer species are occurring in clear altitudinal zones, which enabled us to attribute a certain mean elevational distribution with a specific mean annual temperature (MAT) and mean annual precipitation (MAP) to each species.

## MATERIALS AND METHODS

The botanical material was collected along the highway A‐138 between the Spanish side of the Tunnel d'Aragnouet‐Bielsa (1,675 m a.s.l., 42°43′13.93″N 0°11′52.73″E) and the city of Ainsa (590 m a.s.l., 42°26′0.35″N 0°7′55.08″E) in the province of Huesca, Aragon, Spain. The highway passes through a 1,000‐m elevation gradient with distinct distributional patterns of the investigated conifer species. The high elevations above 1,600 m a.s.l. are dominated by mountain pine (*Pinus uncinata sensu lato* Ramond ex DC), whereas Aleppo pine (*Pinus halepensis* Mill.) becomes the dominant pine species in the lowlands below 800 m a.s.l. The area in between is populated predominantly by Scots pine (*Pinus sylvestris* L.) and Austrian pine (*Pinus nigra sensu lato* J.F. Arnold). However, Scots pine occupies a broader elevational range between 600 m and 1,800 m a.s.l., whereas Austrian pine is limited to altitudes between 690 and 1,400 m a.s.l. All three juniper species are present in the lowlands (400–450 m a.s.l.); however, Common juniper (*Juniperus communis* L.) is more abundant in high elevations. Cade juniper (*Juniperus oxycedrus* L.) occurs up to 1,100 m, and Phoenicean juniper (*Juniperus phoenicea* L.) up to 1,400 m a.s.l. In September 2025, one branch of the sun‐exposed crown was sampled from five dominant individuals for each species. Sampling was conducted, intending to cover the species‐specific elevation range that was easily accessible from the highway. There was less than 3 h between taking the first sampling and the start of the processing of the botanical sample for the heat treatment. We only used healthy needles from one‐year‐old shoots, tested by measuring the photosynthetic quantum use efficiency (*F*_*v*_/*F*_*m*_) with a chlorophyll fluorometer (MINI‐PAM, Walz, Effeltrich, Germany) of all used needles. We defined *F*_*v*_/*F*_*m*_ values between 0.75 and 0.83 to indicate the health of the selected leaves. Per level of temperature treatment, we used five needles, while taking one needle from each of the respective five sampled plant individuals per temperature treatment.

Photosystem II thermotolerance thresholds of *F*_*v*_/*F*_*m*_ were assessed following the modified protocol of Krause *et al*. (2010), described in Kunert *et al*. (2026). Therefore, needles were taped on stiff plastic sheets with perforated medical tape (Transpore™, 3 M™ GmbH, Austria). To prevent sample dehydration, we placed damp paper towels on top of the plastic sheets. The plastic sheets were packed in water‐tight Ziplock bags and immersed in temperature‐controlled water baths for 30 min, regulated by Sous–Vide precision cookers. We opted for eight treatment temperatures ranging from 30 °C to 65 °C, increasing the temperature in steps of 5 °C. After the temperature treatment, the needles were allowed to recover for 24 h at low‐light levels. Before conducting further measurements, the needles were dark acclimated for at least 30 min and *F*_*v*_/*F*_*m*_ was subsequently measured with the chlorophyll fluorometer.

### Estimation of temperature thresholds and climate variables

We used the same log‐logistic curve for the *F*_*v*_/*F*_*m*_ response as described by Kunert *et al*. (2022):

$$F_{v}/F_{m}=c+\frac{d-c}{1+\mathrm{Exp}\left[b\;\log\left(\frac{T}{\text{T}50}\right)\right]\;}$$

where T describes the temperature. Parameter c is *F*_*v*_/*F*_*m*_ of the lower, and d the higher plateau. T50 represents the turning point of the function and reflects the temperature at which *F*_*v*_/*F*_*m*_ declined by 50%. Parameter b is the slope of the curve at T = T50. Response curves were fitted using the ‘modelFit’ function of the ‘drc’ package (Ritz *et al*. 2016) in R. We extracted three different measures, T5, T15 and T50, from the fitted curves to assess the effect of environmental parameters on PSII thermotolerance thresholds, corresponding to the temperatures at which the negative change in *F*_*v*_/*F*_*m*_ is 5%, 15%, or 50% of the maximum value (c, d), respectively. We fitted one curve for each individual measured separately; therefore, the presented PSII thermotolerance thresholds are species means with ±SD. We used the ‘geodata’ package (Hijmans *et al*. 2024) to extract climate data from worldclim (Fick & Hijmans 2017). We extracted MAT and MAP for each location where botanical material was collected. MAT and MAP of all five sample locations per species were averaged for comparison with the PSII thermotolerance thresholds. In addition, we used linear models to determine the effect of elevation and species on the thermal threshold values (T5, T15 and T50) at the individual level. To quantify this, we used elevation and species as fixed effects. Subsequently, we ran a type‐II ANOVA with the ‘car’ package (Fox & Weisberg 2019) and determined the partial effect size with the ‘effectsize’ package (Ben‐Shachar *et al*. 2020). Data analysis was performed using the R program, version 4.5.1 (R Core Team 2025).

## RESULTS

**Fig. 1:**
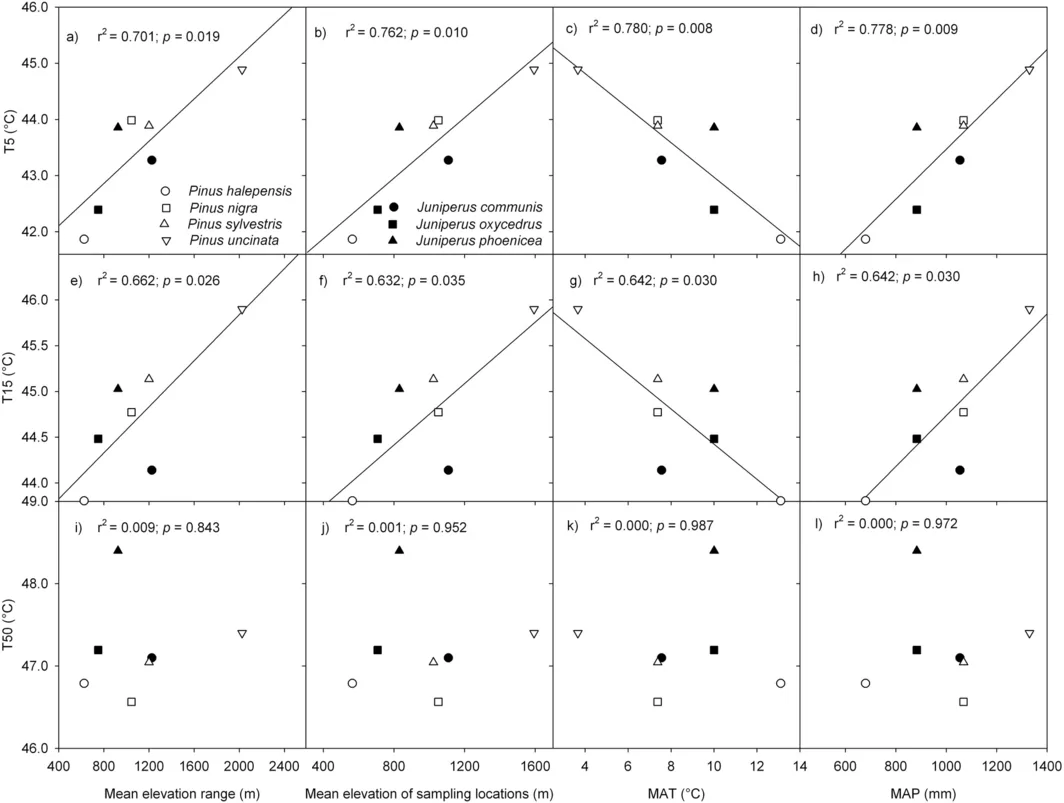
Relationships between the three temperature thresholds T5, T15 and T50 and the mean elevation of a species' distribution in the Pyrenees (a, e, i), the mean elevation of the sampling locations of a given species (b, f, j), the mean annual temperature (MAT) of the sampling area (c, g, k) and the mean annual precipitation (MAP) (d, h, l).

The first two PSII thermotolerance thresholds, namely T5 and T15, were found to exhibit a clear linear increase with altitude. Both species‐specific elevations, the literature value indicating the mean elevation range of the species' distribution (Fig. 1a,e) and the mean elevation of the sampling location (Fig. 1b,f) were correlated with T5 and T15. While mountain pine (*Pinus uncinate*) displayed the highest T5 and T15 of 44.9 °C ± 1.3 °C and 45.9 °C ± 1.2 °C, respectively, Aleppo pine (*Pinus halepensis*) correspondingly showed the lowest values of 41.9 °C ± 3.1 °C and 43.8 °C ± 2.1 °C (Table 1). Similarly, the two PSII thermotolerance thresholds were noted to be correlated with changes in MAT and MAP with elevation. Both T5 and T15 decreased with increasing MAT (Fig. 1c,g) and increased with a rise in MAP (Fig. 1d,h). However, T50 showed no linear relationship with elevation, MAT or MAP (Fig. 1i–l). Elevation was observed to positively influence the thermal thresholds on individual level for T5 and T15. T5 was predicted to increase by 4.1 °C, and T15 by 3.7 °C per 1000 m. Accordingly, elevation explained 15% and 22% of the variance. Contrarily, species identity only accounted for only 8% and 15% of variance. Lastly, T50 values were not impacted by either elevation or species considering threshold values on individual level.

**Table 1:** Summary of the measured thermal tolerance for the seven species; further, the distributional elevation range of each species is provided.

common name	latin name	elevation range^a^		T5	T15	T50
		lowest (m)	highest (m)	mean (°C ± SD)	mean (°C ± SD)	mean (°C ± SD)
Common juniper	*Juniperus communis*	450	2,000	43.3 ± 3.5	44.1 ± 1.8	47.1 ± 1.5
Cade juniper	*Juniperus oxycedrus*	400	1,100	42.4 ± 1.8	44.3 ± 1.2	47.2 ± 1.2
Phoenicean juniper	*Juniperus phoenicea*	450	1,400	43.9 ± 2.5	45.0 ± 2.4	48.5 ± 0.9
Aleppo pine	*Pinus halepensis*	450	800	41.9 ± 3.1	43,8 ± 2.1	46.8 ± 1.0
Austrian pine	*Pinus nigra*	690	1,400	44.0 ± 0.3	44.8 ± 0.2	46.6 ± 0.7
Scots pine	*Pinus sylvestris*	600	1,800	43.9 ± 2.2	45,1 ± 1.6	47.0 ± 1.1
Mountain pine	*Pinus uncinata*	1,600	2,450	44.9 ± 1.3	45.9 ± 1.2	47.4 ± 1.6
Mean				43.3 ± 1.1	44.7 ± 0.7	47.2 ± 0.6

Thermal tolerance traits include the breaking point temperature at which photosystem II (PSII) efficiency declines by 5% (T5), temperature at which PSII efficiency declines by 15% (T15), and temperature at which efficiency is at 50% (T50) of the maximum remains. Thermal tolerance traits represent species means (n = 5) with the standard deviation (SD).

^a^ Distributional range from Villar Pérez *et al*. (1997).

## DISCUSSION

In our study, thermotolerance as a measure of the first significant decline in the quantum use efficiency of the PS II increased with elevation species and individual level. Analogously, these thermotolerance thresholds decreased with increasing MAT and increase with increasing MAP on species level. Thereby, we discovered a trend that shows the exact opposite direction to what was suggested in previous studies in a tropical ecosystem. For example, T_crit_ was discovered to be higher in lower altitudes (Slot *et al*. 2021). In contrast, Hernández *et al*. (2025) observed thermotolerance thresholds, particularly T50, to decline with elevation. In our study, we did not observe any changes in T50 with elevation, MAT or MAP. Regardless of the contradicting results, the explanation delivered by Hernández *et al*. (2025), why T50 might increase with higher altitudes is very appealing and might explain our observations. The authors argue that areas of higher elevation experience extreme diurnal temperature fluctuations, even during the vegetative period. Indeed, frost episodes can even occur during summer in temperate alpine systems, thereby amplifying the overall range of temperature extremes in high altitudes. Accordingly, species growing in higher elevations are exposed to a variety of stressors, namely heat during the day, and counterintuitively frost during night‐time. Therefore, high‐elevation species seem to require adaptations to multiple stressors. This is concurrent with the findings by Levitt (1951), who suggested that the need to adapt to one stressor creates protection mechanisms against other stressors at the same time. Likewise, several studies revealed a leaf co‐adaptation to heat and drought stress in many temperate tree species (*e.g*. Kunert *et al*. 2022; Münchinger *et al*. 2023). Another explanation might be that due to the solar angle, slopy terrain in high elevations receives higher light irradiation levels than comparatively flat lowlands, especially in southern expositions (Strack *et al*. 2021). This results in the induction of a ‘vineyard effect’, as steep slopes can receive up to 30%–40% more solar energy than adjacent flat terrain (Hoppmann *et al*. 2017). This vineyard effect forces leaves to develop higher thermotolerances. We speculate that those adaptations imply that alpine tree vegetation is much more resilient to climate change than previously thought. However, we only found this elevation–thermotolerance relationship to be true for the thermotolerance thresholds indicating an initial decline in the quantum use efficiency of PS II (T5 and T15). Both values, T5 and T15, have been criticized recently, in particular, the ecophysiological importance of these two variables (Perez & Feeley 2020; Winter 2024). The main criticism lies in the potential reversibility of this initial damage. T50 has been suggested to be a more ‘realistic tipping point indicator and measure of irreversible heat damage’ (Winter 2024). Nevertheless, we found significant evidence that T5 and T15 are linked to plant fitness as those values can be attributed to certain environmental conditions on relatively small spatial scales. In conclusion and based on our findings, T5 and T15 may mark important ecological thresholds. T5 and T15 are both highly sensitive and indicate an initial shift from full functionality to photosynthetic impairment, and by these means, describing species distributional patterns. In terms of climate change, our findings suggest that conifer species in higher elevations might be more resistant to increasing temperature stress as previously thought.

## AUTHOR CONTRIBUTIONS

N.K. designed the study. N.K. collected the data. N.K. and E.D. performed the statistical analysis. N.K. wrote the first version of the manuscript. All authors contributed to drafting the final version of the manuscript.

## CONFLICT OF INTEREST

The authors declare no conflict of interest.

## Data Availability

The data that support the findings of this study are openly available in ZENODO at https://zenodo.org/records/20955671.
